# Analysis of risk factors for relapse in pediatric MOG antibody-associated disease: a single-center retrospective cohort study

**DOI:** 10.3389/fneur.2026.1790844

**Published:** 2026-03-26

**Authors:** Xiaohui Min, Guohuan Ying, Xiao Wu, Xiaoyu Liu, Jia Yin, Shuhan Hu, Zihan Fan, Nooraynee Bibi Needah Ginowree, M. Jalal Ud Din, Peng Liu, Gang Zhang

**Affiliations:** 1 Department of Neurology, Children’s Hospital of Nanjing Medical University, Nanjing, China; 2 Department of Pediatrics, Tianjin Union Medical Center, The First Affiliated Hospital of Nankai University, Tianjin, China; 3 Nanjing Medical University, Nanjing, Jiangsu, China; 4 Department of Radiology, Children’s Hospital of Nanjing Medical University, Nanjing, China

**Keywords:** children, clinical phenotype, MOGAD, MRI, relapse

## Abstract

**Objective:**

Myelin oligodendrocyte glycoprotein antibody-associated disease (MOGAD) is an immune-mediated demyelinating disorder of the central nervous system. It has a higher incidence in children than in adults, carries a relatively high risk of relapse with unclear mechanisms, and significantly impacts patient prognosis. This study aimed to investigate the clinical characteristics of MOGAD and identify independent risk factors for relapse, to provide a basis for early intervention and individualized treatment.

**Methods:**

A total of 108 children diagnosed with MOGAD at the Children’s Hospital of Nanjing Medical University between January 2020 and June 2024 were retrospectively enrolled. Clinical, laboratory, and radiological data were collected. Univariate analysis and multivariate logistic regression models were used to screen for risk factors associated with relapse.

**Results:**

The median follow-up time was 37 months, with a relapse rate of 30.6%. The proportion of patients presenting with seizures (54.5% vs. 20.0%, *p* < 0.001) and limb weakness (27.3% vs. 9.3%, *p* = 0.018) at onset was significantly higher in the relapse group compared to the monophasic group. The most common initial clinical phenotype was acute disseminated encephalomyelitis (ADEM) type (38.9%), while optic neuritis (ON) type became predominant at relapse (48.9%). Multivariate analysis identified seizures (OR = 7.155, 95% CI: 2.265–22.604, *p* < 0.001) and limb weakness (OR = 5.157, 95% CI: 1.322–20.117, *p* = 0.018) as independent risk factors for relapse, whereas a normal brain MRI (OR = 0.186, 95% CI: 0.035–0.985, *p* = 0.048) was a protective factor. The relapse group had a higher proportion of patients with high serum MOG antibody titers (≥1:100) (54.5% vs. 32.0%, *p* = 0.027) and elevated cerebrospinal fluid cell counts (>30 × 10^6^/L) (66.7% vs. 44.0%, *p* = 0.03). Patients receiving oral corticosteroids for ≥6 months during the remission phase had a significantly lower relapse rate (42.4% vs. 65.3%, *p* = 0.026).

**Conclusion:**

In pediatric MOGAD, the ADEM phenotype is most common at onset. Seizures and limb weakness at initial presentation are independent risk factors for relapse in children with MOGAD, while a normal brain MRI suggests a lower relapse risk. Prolonging the corticosteroid treatment course during remission (≥6 months) may help reduce the relapse risk. Enhanced follow-up and individualized treatment should be considered for children with high-risk factors. These findings may help clinicians identify high-risk patients and tailor long-term immunosuppressive therapy.

## 1 Introduction

Myelin oligodendrocyte glycoprotein (MOG) is a crucial component of the myelin protein in the central nervous system, located on the surface of myelin sheaths and oligodendrocytes, playing a key role in maintaining myelin structure and function (1, 2). Recent studies have shown that pathogenic MOG antibodies can trigger inflammatory demyelinating lesions in the central nervous system, leading to a condition known as MOGAD. This disease has been recognized as a distinct acquired demyelinating disorder, separate from neuromyelitis optica spectrum disorders (NMOSD) and multiple sclerosis (MS) (3, 4). Epidemiological data indicate that pediatric MOGAD cases account for 25–50% of all new diagnoses (5). The clinical presentation is highly heterogeneous, with approximately 30–50% of affected children experiencing relapses, which may result in irreversible neurological deficits in some patients (6–9).

Currently, research on risk factors associated with relapse in pediatric MOGAD remains relatively limited, and the selection and efficacy of therapeutic agents for preventing MOGAD relapses lack clear evidence-based guidance. Although some studies suggest that factors such as the type of disease onset, antibody titers, and cerebrospinal fluid inflammatory markers may be associated with relapse, there is still no consensus. Therefore, this study, through a large single-center retrospective cohort analysis, aims to systematically characterize the clinical spectrum of pediatric MOGAD and identify independent risk factors for relapse, thereby providing a basis for early intervention and individualized treatment.

## 2 Methods

### 2.1 Study subjects

This study retrospectively collected clinical data from 108 children definitively diagnosed with MOGAD in the Department of Neurology at the Children’s Hospital of Nanjing Medical University between January 2020 and June 2024. This study was approved by the Ethics Committee of the Children’s Hospital of Nanjing Medical University, and informed consent was obtained from the parents or guardians.

*Inclusion criteria*: (1) Age at onset ≤18 years; (2) Meeting the diagnostic criteria proposed by the international MOGAD panel in 2023 (3); (3) Complete clinical data, follow-up time ≥6 months, and good compliance; (4) Voluntary participation in the study with guardian consent and signed informed consent form.

*Exclusion criteria*: (1) Poor family cooperation, lack of collaboration, or missing clinical data; (2) Demyelinating lesions caused by other identified etiologies.

### 2.2 Methods

#### 2.2.1 Clinical data collection

Patient history was collected, including demographic information (sex, age at onset), prodromal events (infection, vaccination), detailed clinical symptoms at onset and relapse, laboratory indicators (serum/cerebrospinal fluid MOG antibody titers, inflammatory markers, autoantibodies, etc.), imaging data (brain/spinal cord/optic nerve MRI, etc.), and treatment information (treatment regimen, drug selection, treatment duration). Patients were followed up for relapse, recording the number of relapses, time of relapse, clinical phenotype, treatment regimen, and prognosis. Neurological functional status was assessed using the Expanded Disability Status Scale (EDSS) and the modified Rankin Scale (mRS).

#### 2.2.2 Definitions and criteria

##### 2.2.2.1 Relapse

The emergence of new neurological symptoms occurring ≥1 month (≥3 months for ADEM phenotype) after the previous attack, accompanied by corresponding changes on imaging studies, lasting >24 h, and excluding other causes (3).

##### 2.2.2.2 Disease course classification

A relapsing course was defined as ≥2 acute attacks during the follow-up period; otherwise, it was classified as a monophasic course (3).

##### 2.2.2.3 Antibody titer

A serum MOG antibody titer ≥1:100 was defined as high titer, and <1:100 as low titer (3).

##### 2.2.2.4 Elevated CSF cell count

Cerebrospinal fluid nucleated cell count >30 × 10^6^/L.

#### 2.2.3 Antibody detection method

Patient serum (1 mL) or paired serum and cerebrospinal fluid (1 mL each) samples were sent externally to EUROIMMUN Medical Laboratory for the detection of serum MOG antibodies using a cell-based assay (CBA). The assay used human embryonic kidney cells (HEK293 cells) transfected with the full-length human MOG gene as the antigen substrate and goat anti-human IgG Fcγ-specific fragment as the secondary antibody. Each serum sample was tested in duplicate simultaneously. Fluorescence images were independently evaluated by two experienced laboratory personnel to ensure the specificity of fluorescent staining.

#### 2.2.4 Statistical methods

Statistical analysis was performed using SPSS 25.0. Measurement data conforming to a normal distribution are expressed as mean ± standard deviation, while non-normally distributed data are expressed as median (interquartile range). Count data are presented as frequency (percentage). Inter-group comparisons were conducted using the *χ*^2^ test, Fisher’s Exact Probability test, or the Wilcoxon Rank-Sum test. Variables with a *p*-value <0.05 in univariate analysis were included in a multivariate logistic regression model to analyze independent risk factors for relapse. A *p*-value <0.05 was considered statistically significant.

## 3 Results

### 3.1 Clinical characteristics

Among the 108 pediatric MOGAD patients, 41 were male (38.0%) and 67 were female (62.0%), with a male-to-female ratio of 1:1.63. The median age at onset was 8.33 years (IQR: 5.33–10.5), with the 5–10 year age group being the most prevalent (55.0%). The median follow-up duration was 37 months (range: 8–62). Thirty-three patients (30.6%) experienced relapses and were assigned to the relapsing course group, while the remaining 75 patients (69.4%) constituted the monophasic course group. There were no statistically significant differences between the two groups regarding gender, age, length of hospital stay, history of prodromal infection, or vaccination (*p* > 0.05). Initial clinical symptoms were diverse, with fever (42/108), visual impairment (35/108), seizures (33/108), headache (26/108), and limb weakness (16/108) being the most common. The proportion of patients presenting with seizures (54.5% vs. 20.0%, *p* < 0.001) and limb weakness (27.3% vs. 9.3%, *p* = 0.018) at onset was significantly higher in the relapse group compared to the monophasic group (Table 1). The initial clinical phenotype was most frequently ADEM type (38.9%) in both groups, followed by ON type (23.1%) and encephalitis type (20.4%) (Table 2). There was no statistically significant difference in the initial clinical phenotypes between the two groups. Among the 47 relapse events recorded in the relapsing course group, the ON type was the most common (48.9%, 23/47), followed by the ADEM type (29.8%, 14/47).

**Table 1:** Initial clinical presentations of 108 pediatric MOGAD patients and a comparison between the two groups.

Clinical manifestations [*n* (%)]	Total *n* = 108	Monophasic course group *n* = 75	Relapsing course group *n* = 33	*x*^2^ value	*p*-value
Fever	42 (38.9)	29 (38.7)	13 (39.4)	0.005	0.943
Convulsions	33 (30.6)	15 (20.0)	18 (54.5)	12.889	<0.01**
Headache	26 (24.1)	19 (25.3)	7 (21.2)	0.213	0.644
Dizziness	10 (9.3)	6 (8.0)	4 (12.1)	0.103	0.749
Vomiting	8 (7.4)	5 (6.7)	3 (9.1)	0.002	0.965
Impaired consciousness	8 (7.4)	4 (5.3)	4 (12.1)	0.709	0.4
Visual disturbances	35 (32.4)	28 (37.3)	7 (21.2)	2.719	0.099
Limb weakness	16 (14.8)	7 (9.3)	9 (27.3)	4.509	0.018*
Ataxia	9 (8.3)	7 (9.3)	2 (6.1)	0.036	0.85
Urinary and fecal dysfunction	9 (8.3)	5 (6.7)	4 (12.1)	0.321	0.571
Mental and behavioral abnormalities	3 (2.8)	3 (4.0)	0 (0)	—	0.551
Cognitive changes	2 (1.9)	2 (2.7)	0 (0)	—	1
Language abnormalities	4 (3.7)	4 (5.3)	0 (0)	0.638	0.424
Sensory abnormalities	1 (0.9)	1 (1.3)	0 (0)	—	1

*p < 0.05,**p < 0.01.

**Table 2:** Initial clinical phenotypes of 108 pediatric MOGAD patients and comparison between the two groups.

Clinical phenotype [*n* (%)]	Total *n* = 108	Monophasic course group *n* = 75	Relapsing course group *n* = 33	*x*^2^ value	*p*-value
ADEM	42 (38.9)	26 (34.7)	16 (48.5)	1.841	0.175
ON	25 (23.1)	19 (25.3)	6 (18.2)	0.659	0.417
Encephalitis type	22 (20.4)	14 (18.7)	8 (24.2)	0.439	0.507
NMOSD	6 (5.6)	5 (6.7)	1 (3.0)	0.092	0.761
TM	2 (1.9)	2 (2.7)	0 (0)	—	1
Other phenotypes	11 (10.2)	9 (12.0)	2 (6.1)	0.354	0.552

### 3.2 Laboratory and imaging findings

**Figure 1:**
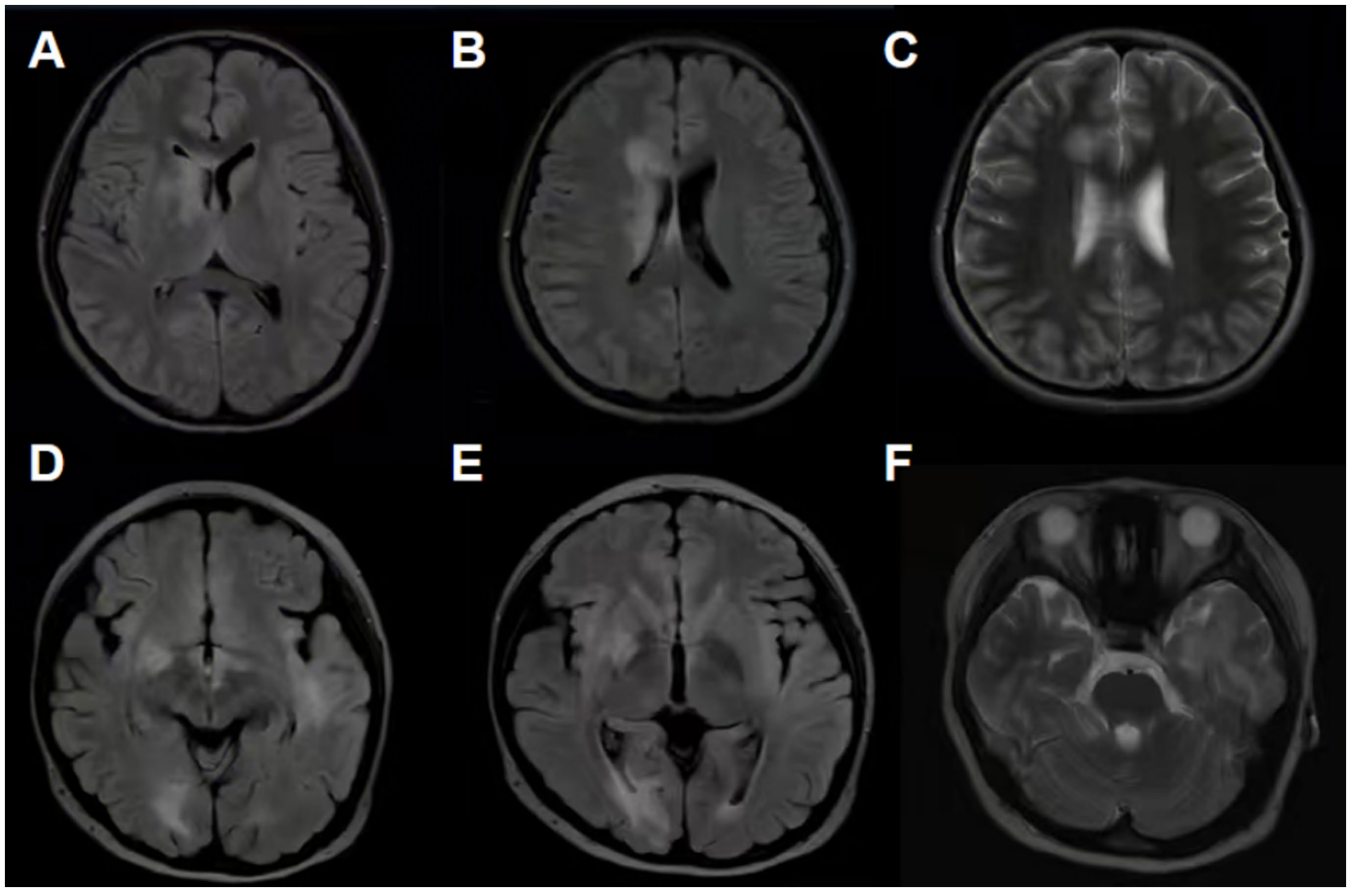
**(A–C)** 11 year-old girl; **(D–F)** 12 year-old girl. **(A)** FLAIR sequence shows patchy hyper-intense signals in the right basal ganglia and the genu of the corpus callosum. **(B)** FLAIR sequence shows patchy hyper-intense signals adjacent to the right lateral ventricle and in the body of the corpus callosum. **(C)** T2 sequence shows patchy hyper-intense signals in the deep cerebral white matter of the right frontal lobe. **(D)** FLAIR sequence shows patchy hyper-intense signals in both temporal lobes and the right occipital lobe. **(E)** FLAIR sequence shows patchy hyper-intense signals in the right basal ganglia and the right occipital lobe. **(F)** T2 sequence shows extensive patchy hyper-intense signals in the left temporal lobe.

At initial onset, all MOGAD patients underwent serum MOG antibody testing and cerebrospinal fluid (CSF) examination. The relapse group had a higher proportion of patients with high serum MOG antibody titers (≥1:100) (54.5% vs. 32.0%, *p* = 0.027) and elevated CSF cell counts (nucleated cell count >30 × 10^6^/L) (66.7% vs. 44.0%, *p* = 0.03). There was no significant difference in the CSF MOG antibody positivity rate between the two groups. All 108 patients underwent brain MRI during the acute phase, with abnormal signals detected in 84 patients (77.8%, 84/108). The primary sites of involvement were white matter (41/84), deep gray matter (33/84), cortex (32/84), cerebellum (21/84), and brainstem (14/84) (Figure 1). A normal brain MRI was more common in the monophasic group (29.3% vs. 6.1%, *p* = 0.007). A total of 87 patients underwent spinal MRI during the acute phase, with 33 showing spinal cord involvement, most commonly affecting the thoracic cord (14/33) and cervical cord (11/33) (Table 3).

**Table 3:** Laboratory and imaging findings of 108 pediatric MOGAD patients: a comparison between the two groups.

Variable	Total *n* = 108	Monophasic course group *n* = 75	Relapsing course group *n* = 33	*x*^2^ value	*p*-value
Serum MOG antibody titer [*n* (%)]					
<1:100	66 (61.1)	51 (68.0)	15 (45.5)	4.901	0.027*
≥1:100	42 (38.9)	24 (32.0)	18 (54.5)		
CSF examination [*n* (%)]					
MOG antibody positive	38 (35.2)	26 (34.7)	12 (36.4)	0.029	0.865
Elevated cell count (>30 × 10^6^/L)	55 (50.9)	33 (44.0)	22 (66.7)	4.711	0.03*
Elevated protein	22 (20.4)	12 (16.0)	10 (30.3)	2.89	0.89
Brain MRI [*n* (%)]	108	75	33		
No significant abnormalities	24 (22.2)	22 (29.3)	2 (6.1)	7.181	0.007**
Cortical	32 (29.6)	22 (29.3)	10 (30.3)	0.01	0.919
White matter	41 (38.0)	27 (36.0)	14 (42.4)	0.402	0.526
Deep gray matter	33 (30.6)	22 (29.3)	11 (33.3)	0.173	0.678
Cerebellum	21 (19.4)	15 (20.0)	6 (18.2)	0.048	0.826
Brainstem	14 (13.0)	10 (13.3)	4 (12.1)	0.3	0.863
Spinal MRI	87	57	30		
Cervical cord	11 (12.6)	6 (10.5)	5 (16.7)	0.23	0.631
Thoracic cord	14 (16.1)	11 (19.3)	3 (10.0)	0.664	0.415
Lumbar cord	4 (4.6)	4 (7.0)	0 (0)	0.897	0.344
Entire spinal cord	9 (10.3)	6 (10.5)	3 (10.0)	0	1

*p < 0.05,**p < 0.01.

### 3.3 Treatment and prognosis

For acute-phase treatment of the initial onset, all patients received immunotherapy: 56.5% (*n* = 61) received a combination of intravenous methylprednisolone (IVMP) and intravenous immunoglobulin (IVIG), 26.9% (*n* = 29) received IVMP alone, 12.0% (*n* = 13) received dexamethasone combined with IVIG, while the remaining patients received IVIG/dexamethasone alone or plasma exchange. Clinical symptoms improved significantly after treatment, with no significant difference in acute-phase treatment regimens between the two groups (*p* > 0.05). A mRS score of 3–5 is commonly used as a disability criterion. Defining patients with an mRS score of 3–5 as having severe condition at admission, 31 patients were classified as severe at admission, 14 of whom experienced relapse. This suggests an association between an admission mRS score ≥3 and an increased risk of relapse (42.4% vs. 22.7%, *p* = 0.037). All 108 patients received sequential oral corticosteroid therapy. During follow-up, a total of 63 patients received oral corticosteroids for ≥6 months during the remission phase. The proportion of patients with an oral corticosteroid course ≥6 months was significantly lower in the relapse group compared to the monophasic group (42.4% vs. 65.3%, *p* = 0.026) (Table 4).

**Table 4:** Treatment, prognosis, and intergroup comparison in 108 pediatric MOGAD patients.

Variable	Total *n* = 108	Monophasic course group *n* = 75	Relapsing course group *n* = 33	*Z*/*x*^2^	*p*-value
Acute phase treatment regimen [*n* (%)]					
IVMP	29 (26.9)	21 (28.0)	8 (24.2)	0.165	0.685
IVIG	2 (1.9)	2 (2.7)	0 (0)	—	1
IVMP + IVIG	61 (56.5)	42 (56.0)	19 (57.6)	0.023	0.879
Dexamethasone + IVIG	13 (12.0)	8 (10.7)	5 (15.2)	0.115	0.735
Dexamethasone	3 (2.8)	3 (4.0)	0 (0)	—	0.551
Plasma exchange	2 (1.9)	1 (1.3)	1 (3.0)	—	0.52
Duration of remission phase hormone therapy [*n* (%)]					
<6 months	45 (41.7)	26 (34.7)	19 (57.6)	4.948	0.026*
≥6 months	63 (58.3)	49 (65.3)	14 (42.4)		
EDSS score [median (P25, P75)]					
EDSS at admission	2 (1.5, 3.5)	1.5 (1.5, 3.0)	2.5 (1.5, 4.0)	−1.606	0.108
EDSS at admission	0 (0, 1)	0 (0, 1)	0 (0, 1.5)	−1.929	0.054
mRS score [median (P25, P75)]					
mRS at admission	2 (1, 3)	2 (1, 2)	2 (1, 3)	−0.514	0.607
mRS at discharge	0 (0, 0)	0 (0, 0)	0 (0, 1)	−1.253	0.21
Admission mRS ≥ 3	31 (28.7)	17 (22.7)	14 (42.4)	4.371	0.037*

*p < 0.05,**p < 0.01.

### 3.4 Logistic regression analysis of risk factors for relapse

In this study, whether MOGAD relapsed was used as the dependent variable. Predictors found to be statistically significant (*p* < 0.05) in the univariate analysis were included as independent variables in a multivariate logistic regression analysis to establish a relapse risk prediction model. These variables included: initial clinical presentation with seizures, initial clinical presentation with limb weakness, serum MOG antibody titer ≥1:100, elevated CSF cell count (>30 × 10^6^/L), normal brain MRI, oral corticosteroids during remission ≥6 months, and admission mRS score ≥3.

After adjusting for confounding factors, the regression analysis results indicated that patients with an initial clinical presentation of seizures (OR = 7.155, 95% CI: 2.265–22.604, *p* < 0.001) or limb weakness (OR = 5.157, 95% CI: 1.322–20.117, *p* = 0.018) had an increased risk of relapse. Conversely, patients with a normal brain MRI (OR = 0.186, 95% CI: 0.035–0.985, *p* = 0.048) (Table 5) had a decreased risk of relapse. These were identified as independent prognostic risk factors.

**Table 5:** Multivariate logistic regression analysis of risk factors for relapse in MOGAD.

Variable	Regression coefficient	Standard error	Wald	*p*	OR	OR 95% CI	
						Lower	Upper
Seizures	1.968	0.587	11.243	<0.01*	7.155	2.265	22.604
Limb weakness	1.64	0.694	5.58	0.018*	5.157	1.322	20.117
Serum MOG-Ab Titer ≥1:100	1.062	0.553	3.687	0.055	2.891	0.978	8.545
Elevated CSF cell count	1.048	0.57	3.384	0.066	2.852	0.934	8.709
No significant abnormalities on brain MRI	−1.682	0.85	3.912	0.048*	0.186	0.035	0.985
Oral corticosteroids ≥6 months in remission phase	−0.624	0.563	1.228	0.268	0.536	0.178	1.615
mRS ≥ 3 at admission	0.755	0.585	1.664	0.197	2.128	0.675	6.704

*p < 0.05,**p < 0.01.

## 4 Discussion

This study retrospectively analyzed clinical data from 108 pediatric patients with MOGAD and found a relapse rate of approximately 30.6%, which aligns with previously reported international rates for pediatric MOGAD (approximately 30–50%) (10, 11). This further confirms the high tendency for relapse of this disease in the pediatric population. Based on the disease course characteristics, the 108 patients were categorized into monophasic and relapsing course groups. A comparative analysis of epidemiological features, clinical manifestations, laboratory results, MRI imaging characteristics, treatment regimens, and efficacy between the two groups revealed potential factors that may influence disease relapse.

Among the 108 pediatric MOGAD patients included in this study, 41 were male and 67 were female, resulting in a male-to-female ratio of 1:1.63, consistent with previous literature reports (12). Some studies have found that a history of prodromal infection, particularly respiratory infection, is an independent risk factor for relapse in pediatric MOGAD (13). This study found that the proportion of patients presenting with seizures and limb weakness at onset was significantly higher in the relapsing course group compared to the monophasic group. Further multivariate logistic regression analysis indicated that these were independent risk factors for relapse. Domestic studies also indicate that MOGAD patients with epileptic seizures have a significantly higher relapse rate than those without (14). Therefore, for children presenting clinically with seizures or limb weakness, brain MRI should be promptly performed, and MOG antibody testing should be considered to rule out MOGAD. For children whose initial presentation includes seizures or limb weakness, enhanced follow-up and immune monitoring are recommended to reduce the risk of relapse and improve prognosis.

Previous studies indicate that the main clinical phenotypes in children are ADEM, ON, myelitis, and encephalitis types, with ADEM being the most common (15). In this study, the initial clinical phenotype was ADEM in 38.9% of cases, consistent with this. Some studies suggest that patients presenting with ON at onset have a higher relapse risk than those presenting with transverse myelitis (TM) (16), but this study did not find that different clinical phenotypes influenced relapse. Notably, the main clinical phenotype changed during subsequent relapses in the relapsing course group, with ON (48.9%) becoming predominant, followed by ADEM (29.8%) and encephalitis (14.9%), differing from the initial predominance of ADEM. Previous international studies have also indicated that MOGAD exhibits diverse clinical manifestations, and may present with different phenotypes across various attack phases (17, 18). The results of this study further confirm the diversity of clinical phenotypes in pediatric MOGAD and the potential for phenotype changes upon relapse, suggesting the need in clinical practice to monitor the dynamic changes in MOGAD to optimize diagnostic and therapeutic strategies.

Positive MOG antibody detection is a core criterion for MOGAD diagnosis. Currently, there is no international consensus on whether high-titer serum MOG antibodies at the initial disease stage or persistent positivity can serve as potential biomarkers for predicting relapse risk in MOGAD (19). This study found that high serum MOG antibody titer (≥1:100) was significantly associated with relapse risk. However, as dynamic changes in antibody titers were not regularly monitored, further research is needed to clarify its correlation with relapse and disease severity. In this study, all patients underwent CSF examination at initial onset. The positivity rate for CSF MOG antibodies was 35.2%, significantly lower than serum antibody titers, consistent with the current view that MOG antibodies are primarily produced in the blood (20). There was no significant difference in CSF MOG antibody positivity between the two groups, indicating no clear association with relapse risk. However, elevated CSF cell count (>30 × 10^6^/L) was more common in the relapsing course group, suggesting it may be associated with a tendency to relapse.

Magnetic resonance imaging is an important examination tool for MOGAD patients. In this study, 84 patients had abnormal brain MRI findings, commonly involving white matter (48.8%), cortex (38.1%), and deep gray matter (39.3%), with lesions often being multifocal and poorly defined. This MRI presentation may be related to the pathological mechanism of MOGAD, namely immune-mediated inflammation leading to myelin damage and demyelination. A significantly higher proportion of patients in the monophasic group had a normal initial brain MRI compared to the relapsing group, suggesting that the absence of abnormalities on the initial brain MRI is associated with a lower risk of relapse. A typical feature on spinal MRI is longitudinally extensive TM (LETM), defined as a continuous lesion spanning ≥3 vertebral segments (21), commonly involving the cervical and thoracic cord. These lesions often appear as central hyper-intensity on T2-weighted images, sometimes with an H-shaped distribution. In this study, the most common site of spinal cord involvement was the thoracic cord (42.4%), followed by the cervical cord (33.3%), with some patients showing involvement of the entire spinal cord, consistent with previous reports.

Current treatment regimens for MOGAD primarily refer to the 2020 European guidelines for pediatric MOGAD (22). The relationship between different treatment choices and relapse remains unclear. Some studies found that treatment with IVMP alone during the acute phase yielded good short-term efficacy, but many patients subsequently experienced clinical relapse (23), suggesting that acute-phase treatment with IVMP alone might be a factor influencing relapse. However, in this study, there was no statistical difference in acute-phase treatment regimens between the monophasic and relapsing groups. This study found that patients with an admission mRS score ≥3 had a significantly higher relapse rate, indicating that baseline disability level is an important predictor of relapse.

Although there is currently no internationally unified guideline for MOGAD remission-phase treatment, an increasing number of studies in recent years support the role of corticosteroids or immuno-suppressants in preventing relapse. Insufficient duration of maintenance therapy may fail to completely control the residual inflammatory microenvironment, thereby increasing relapse risk. Rapid corticosteroid tapering may trigger immune rebound, whereas slow tapering may reduce autoantibody regeneration by stabilizing inflammatory cell balance. By analyzing follow-up data from the 108 patients, this study found that patients who received oral corticosteroid therapy for ≥6 months after the first episode had a significantly lower relapse rate than those with shorter courses, suggesting that extending the corticosteroid treatment period may reduce relapse risk. These results further support the recommendation of extending corticosteroid therapy to ≥6 months for children at high risk of relapse, with adjustments based on dynamic biomarker monitoring. However, simply prolonging the course of corticosteroid therapy does not fully account for the observed differences in prognosis. We observed that some patients still experienced relapse even after receiving corticosteroids for ≥6 months, suggesting the involvement of other immunomodulatory mechanisms or genetic susceptibility factors.

As a single-center clinical study, this research has certain limitations. The relatively limited sample size inevitably introduces selection bias. Existing literature indicates that adult patients with MOGAD may have a higher relapse rate, with ON being the predominant clinical phenotype, which may be related to differences in immune background, antibody clearance capacity, and corticosteroid metabolism across age groups (24). Therefore, future cross-age comparative studies are warranted to clarify the differences in relapse risk factors between pediatric (<18 years) and adult (>18 years) MOGAD patients. Multicenter, large-sample longitudinal studies are needed to more comprehensively investigate the risk factors for MOGAD relapse and provide more reliable evidence-based support for the development of relapse prevention strategies.

## Data Availability

The original contributions presented in the study are included in the article/supplementary material, further inquiries can be directed to the corresponding authors.
